# Splenic artery pseudoaneurysm; a cause or consequence: a case report

**DOI:** 10.1186/s13256-024-04581-5

**Published:** 2024-05-20

**Authors:** Aymen Abbas, Fatma Mahmoud, Waqar Gaba

**Affiliations:** Department of Medicine, Sheikh Khalifa Medical City, Abu Dhabi, UAE

**Keywords:** Pseudoaneurysm, Pancreatitis, Gastrointestinal bleeding

## Abstract

**Background:**

Splenic artery pseudoaneurysm is a rare complication of recurrent pancreatitis usually presenting as an incidental finding on abdominal computed tomography.

**Case presentation:**

We present the case of a 66-year-old north African male with a known history of previous pancreatitis who presented with upper gastrointestinal bleeding along with recurrent epigastric pain for 3 days. Investigations did not reveal any particular pancreatitis etiology. Computed tomography of the abdomen with contrast showed splenic artery pseudoaneurysm along with findings suggestive of acute pancreatitis. Upper and lower endoscopies failed to identify gastrointestinal the bleed source. The patient underwent intervention radiology embolization of the aneurysm sac with multiple coils via right retrograde common femoral artery–celiac access. The patient was discharged with a plan for capsule endoscopy in outpatient setting.

**Conclusion:**

Splenic artery pseudoaneurysm is a life-threatening complication and carries a high mortality rate if left untreated. Prompt identification through various imaging modalities, followed by urgent intervention, is crucial to avoid adverse outcomes.

## Introduction

Aneurysmal degeneration of the visceral branches of the abdominal aorta is a rare and potentially life-threatening disease entity that can develop as a complication of acute or chronic pancreatitis. The augmented use of ultrasonography and cross-sectional body imaging for intraabdominal pathology has raised the prevalence and incidental identification of visceral artery pseudoaneurysms (VAPA) [1]. Although they might remain asymptomatic, they continue to carry a risk of rupture. Current open surgical therapeutic options for VAPA include aneurysm resection with revascularization, aneurysm ligation, or end-organ resection (that is, splenectomy). Endovascular approaches to managing VAPA offer an alternative to conventional open surgery with the benefit of low procedural morbidity and mortality [1].

## Case description

A 66-year-old north African male presented with a past medical history including essential hypertension, stage 3A chronic kidney disease with a baseline glomerular filtration rate (GFR) of 55 ml/minute/1.73 m^2^, dyslipidemia, benign prostatic hyperplasia, history of lower limb varicose veins, and deep venous thrombosis for which he was not on anticoagulation. Past medical history was also significant for hospital admission a year ago due to acute pancreatitis that resolved after conservative treatment without a clear etiology after the initial investigations. This was followed by another episode of acute pancreatitis 1 month prior to this presentation for which he was admitted for 3 days and managed conservatively. A follow-up computed tomography imaging of the abdomen at that time showed a new finding of a splenic artery dilatation, suggesting splenic artery pseudoaneurysm contained within a cystic lesion, which likely represents a pseudocyst.

**Fig. 1:**
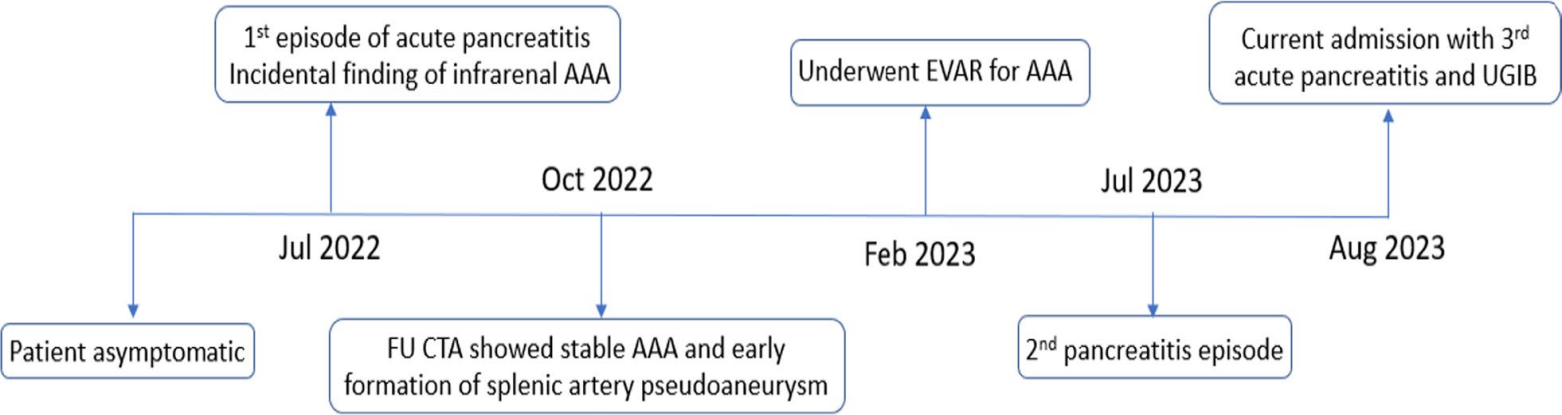
Timeline of recurrent pancreatitis attacks

Surgical history was significant for endovascular aneurysm repair (EVAR) for symptomatic abdominal aortic aneurysm 6 months ago, recurrent bilateral inguinal hernia requiring surgical repair, and intestinal obstruction requiring surgery intervention. He is an ex-smoker, and there was no alcohol abuse history. History was negative for new medication or herbal supplement, recent endoscopic retrograde cholangiopancreatography (ERCP) procedure, or abdominal trauma. Figure 1, showing the timeline, illustrates the sequence of events regarding his recurrent pancreatitis.

The patient presented with severe recurrent epigastric pain that was worsening in the past 3 days. It was associated with four episodes of hematemesis (around 50 mL of blood each time), nausea, and generalized fatigue. On presentation, the patient was afebrile, blood pressure was 152/100 mmHg, pulse rate was 75 beats/minute, respiratory rate was 20 breaths/minute, and oxygen saturation was 96% on room air. Physical examination revealed a soft abdomen but with severe epigastric tenderness. There was no organomegaly nor signs of chronic liver disease. His cardiopulmonary examination was unremarkable. Laboratory studies showed a creatinine level of 123 mmol/L (at baseline), lipase of 1822 IU/L, normal electrolytes, liver function test, and coagulation panel, white blood cell count of 8.3 × 10^9^/L, hemoglobin of 14.8 g/dL, and platelet count of 270 × 10^9^/L. The C-reactive protein was 21 mg/L. Venous blood gases showed pH (potential hydrogen) of 7.39, HCO_3_ of 27 mmol/L, and lactic acid of 3.3 mmol/L. Results showed a triglyceride level of 1.18 mmol/L, immunoglobulin G4 (IgG4) of 0.74 g/L, and corrected calcium level of 2.21 mmol/L (Table 1).

**Table 1:** Initial laboratory studies

Variable	Reference range	Result
Hemoglobin (g/dl)	11.6–14.8	14.8
White blood cell count (per μL)	4.5–11.0	8.3 × 10^9^/L
Neutrophils (%)	0.0–2.5	69.4
Lymphocytes (%)	16.5–49.5	20.1
Monocytes (%)	2.0–10.0	8
Eosinophil (%)	0.0–8.5	0.6
Platelet count (per μL)	140,000–400,000	270,000
AST (IU/L)	≤ 32	21
ALT (IU/L)	≤ 33	10
Albumin (g/L)	25–52	35
Sodium (mmol/L)	135–145	135
Potassium (mmol/L)	3.6–4.8	4.1
Chloride (mmol/L)	101–108	104
Calcium corrected (mmol/L)	2.23–2.58	2.21
Creatinine (μmol/L)	61–106	123
Urea nitrogen (mmol/L)	2.80–8.10	2.9
C‐reactive protein (mg/dL)	≤ 5	21
Procalcitonin (ng/mL)	≤ 0.50	0.05
Lactic acid VBG (mmol/L)	0.5–2.2	3.3
Triglyceride (mmol/L)	< 1.69	1.18
IgG4 g/L	0.052–1.250	0.74

He was admitted as a case of upper gastrointestinal bleeding and acute pancreatitis for investigations.

**Fig. 2:**
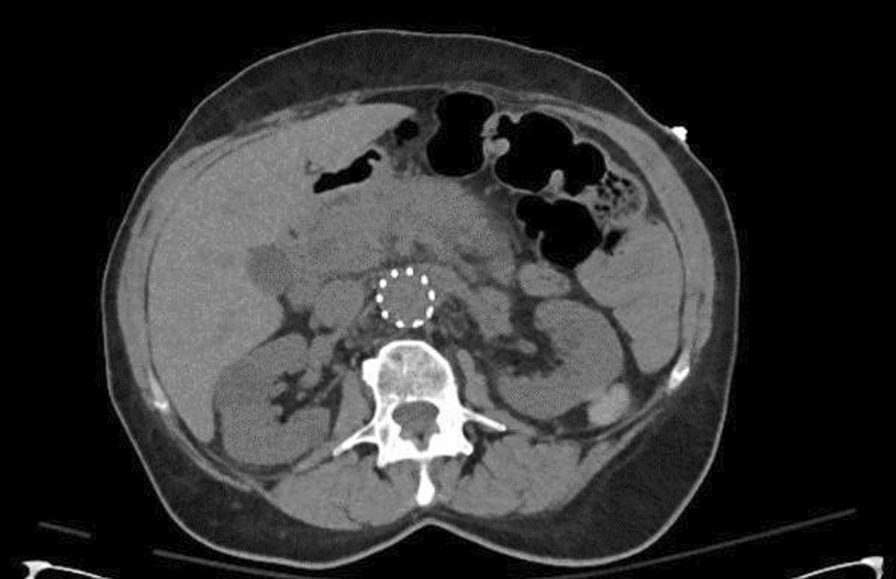
Computed tomography of the abdomen showing acute pancreatitis features

**Fig. 3:**
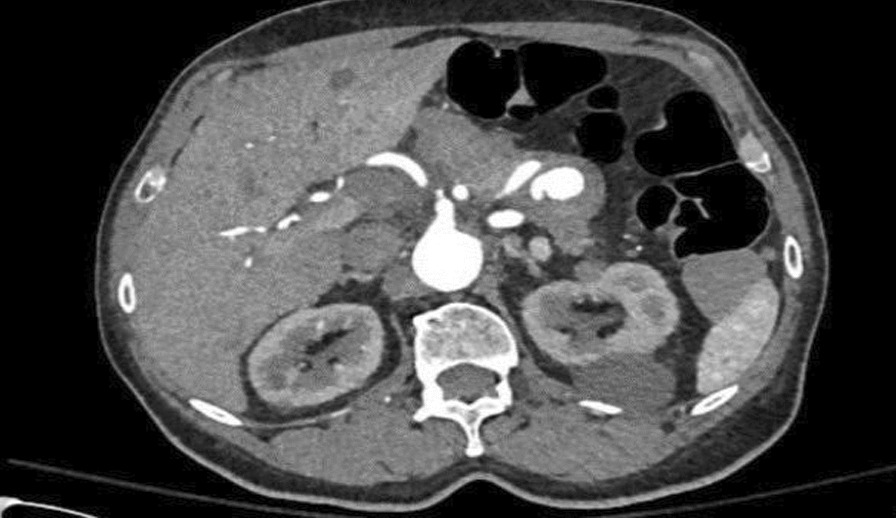
Computed tomography of the abdomen showing splenic artery pseudoaneurysm

**Fig. 4:**
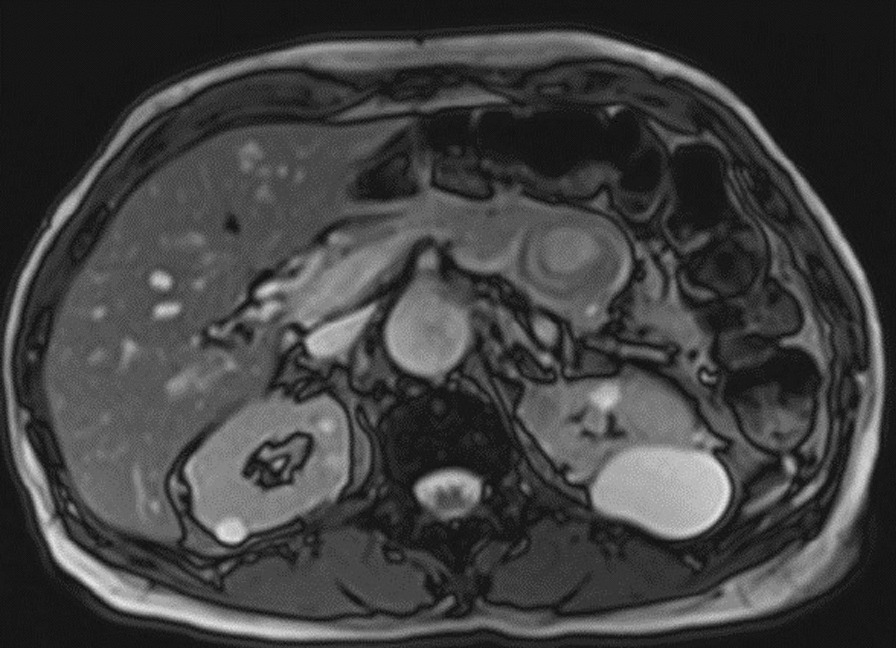
Magnetic resonance cholangiopancreatography showing pseudoaneurysm in the body of the pancreas

Computed tomography angiogram (CTA) done on admission ruled out fistula and showed stable abdominal aortic and iliac artery stents with no features that suggest active extravasation, along with edematous pancreatic head and proximal body, features representing underlying pancreatitis changes (Fig. 2). It also redemonstrated the incidental finding of splenic artery pseudo-aneurysm within a cystic lesion likely representing pseudocyst size 3.6 × 3.7 cm with no active bleeding (Fig. 3). Magnetic resonance cholangiopancreatography (MRCP) showed thin-walled gallbladder with no filling defect or stone, and no common bile duct CBD dilatation, stone, or stricture. It also showed irregular dilatation of the distal pancreatic duct down to a 3.6-cm pseudoaneurysm in the body of the pancreas (Fig. 4). Pancreatitis was managed conservatively with intravenous fluid hydration and symptom-based therapy with improvement in the patient’s clinical condition and symptoms.

**Fig. 5:**
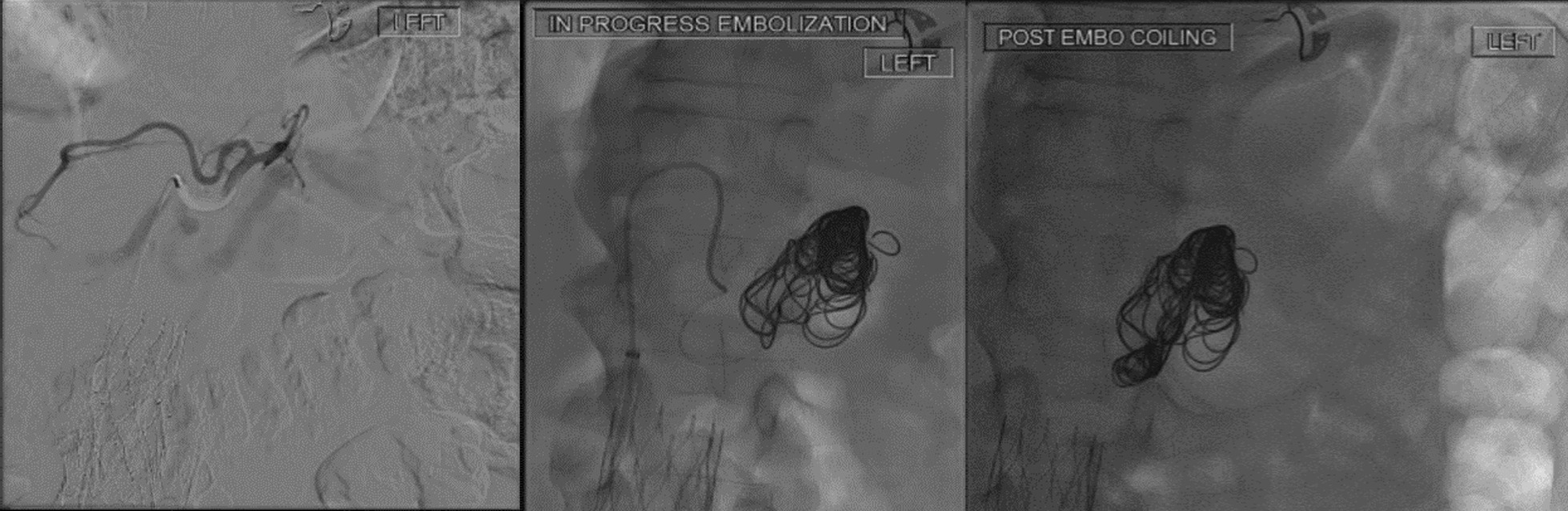
Pre-, during, and post-coiling embolization of splenic artery pseudoaneurysm

The patient was reviewed by the vascular surgery team, and 2 days after admission he underwent intervention radiology (IR) embolization of the aneurysm sac with multiple coils via right retrograde common femoral artery–celiac access (Fig. 5).

Investigations for the upper gastrointestinal bleeding including an esophagogastroduodenoscopy (EGD), which showed normal esophageal mucosa, gastroesophageal junction at 36 cm from the incisors with Hill grade II hiatal hernia. The stomach had mild to moderate erythema in the body and antrum with otherwise no evidence of erosions, ulcers, or masses. The duodenum showed moderate duodenitis seen in D1 with a normal second part of the duodenum. No signs or stigmata of recent bleeding or blood were noticed throughout the examination.

EGD with push enteroscopy was repeated again 5 days after admission due to evidence of ongoing bleeding; however it failed to show a source of bleeding, including the proximal jejunum, which showed normal mucosa with no evidence of erosions, ulcers, or masses. Sigmoidoscopy and full colonoscopy were eventually done and showed internal hemorrhoids with multiple diverticulae in the sigmoid and descending colon filled with altered blood, but no active bleeding was noted.

Further investigations for gastrointestinal (GI) bleeding included insertion of capsule endoscopy for follow-up in the outpatient setting. Follow-up imaging showed stable aneurysm size with no extravasation.

## Discussion

Visceral artery pseudoaneurysms are relatively rare, with a reported incidence of 0.1–0.2%, although the true incidence is not known since many are asymptomatic [2]. The splenic artery and hepatic artery are most commonly involved, but only 157 splenic artery pseudoaneurysms (SAPs) identified in the literature. In contrast to true aneurysms, which involve all three layers (intima, media, and adventitia) of an arterial wall, pseudoaneurysms typically involve only the intima and media. Unlike true aneurysms, SAPs carry a much higher risk of rupture [3]. Pancreatic disease (chronic pancreatitis, acute pancreatitis, pancreatic pseudocyst) was the leading cause in 52% of cases followed by abdominal trauma (29%), and more rare causes included peptic ulcer disease (2%) and postoperative complication (3%) [4]. Our patient likely developed splenic artery pseudoaneurysm within a cystic lesion representing pseudocyst. The reported mechanism of SAP is autodigestion of the splenic artery due to pancreatic proteolytic enzymes, which results in structural disruption of the elastic tissue planes in the vessel wall and subsequent pseudoaneurysm formation [5]. It is also hypothesized that a long-standing pseudocyst can induce SAP due to vascular erosion from enzymes within the pseudocyst [6].

Presentation of splenic artery pseudoaneurysm varies from an incidental finding to acute hemodynamic collapse in some patients. Diagnosis is best established with either CT or ultrasound. The diagnosis of SAP is challenging in the presence of a peripancreatic fluid collection or pseudocyst, where CT of the abdomen can miss smaller sized pseudoaneurysm [7].

Our patient had a previous episode of acute pancreatitis managed at another facility around 1 year ago as evidenced by elevated lipase and CT abdomen showing bulky body and tail of pancreatitis (July 2022). He eventually had another CT angiography of the abdomen and pelvis (Nov 2022) to evaluate the abdominal aortic aneurysm. After review of the images by our vascular surgery team, a small splenic artery pseudoaneurysm was visible but missed on the report.

Transcatheter embolization is one of the interventions used to manage splenic artery pseudoaneurysm. For this procedure, the artery from which the pseudoaneurysm originates is selectively catheterized and embolized with coils just distal and proximal to the lesion. This effectively excludes the aneurysm from the circulation and enables thrombosis. The advantage of this approach is a high success rate, minimal rate of complication, and shorter hospital stay as compared with surgical exploration. Transcatheter embolization of splenic artery pseudoaneurysms has been proven to be a safe and effective and may induce less morbidity than open surgery on the basis of a study by Loffroy [8]. Our patient underwent this procedure with satisfactory results and significant reduction in flow.

Other interventions for the management of ruptured and intact pseudoaneurysms include splenectomy with or without distal pancreatectomy, which has high success rate and ligation alone but with a high failure rate (43%) [4].

Workup for pancreatitis etiology did not relive the underlying cause. The patient had no gallbladder stones, no history of alcohol intake, normal calcium level, and normal IgG4 level and was not on medications that are known to induce pancreatitis. It is believed that he had late-onset idiopathic pancreatitis that was complicated by splenic artery pseudoaneurysm in pseudocyst, which was diagnosed incidentally on CT angiography and magnetic resonance cholangiopancreatography. Fortunately, the patient did not have associated complications, which include rupture and bleeding into the associated pseudocyst. Other complications would include hemorrhage into the pancreatic duct leading to hemosuccus pancreatitis, which can present with hematemesis, hematochezia, or melanotic stool [9].

## Conclusion

Although the pathophysiology is not fully understood, there is a strong association between pancreatitis and pseudoaneurysms of the splenic artery. Such a complication is rare but life threatening, and has a high mortality rate if left untreated. Additionally, the presence of infection and chronic disease can worsen the prognosis of this very serious problem. Most pseudoaneurysms are asymptomatic and identified as incidental findings during imaging but can occasionally cause symptoms when extravasation or internal bleeding occurs. However, early recognition by different imaging modalities, including ultrasound (US) and CT, followed by urgent intervention is crucial to avoid further cataphoric outcomes. The minimally invasive interventional radiology approach remains the mainstay in management. Further studies comparing different treatment approaches outcomes are highly needed in the future.

## Data Availability

Data supporting this study are included within the article.
